# Biosynthesis and Antimicrobial Evaluation of Zinc Oxide Nanoparticles Using *Chlorella vulgaris* Biomass against Multidrug-Resistant Pathogens

**DOI:** 10.3390/ma16020842

**Published:** 2023-01-15

**Authors:** Mohammad Hossein Morowvat, Kimia Kazemi, Maral Ansari Jaberi, Abbas Amini, Ahmad Gholami

**Affiliations:** 1 Biotechnology Research Center, Shiraz University of Medical Sciences, Shiraz P.O. Box 71468-64685, Iran; 2 Pharmaceutical Sciences Research Center, Shiraz University of Medical Sciences, Shiraz P.O. Box 71468-64685, Iran; 3 Department of Pharmaceutical Biotechnology, School of Pharmacy, Shiraz University of Medical Sciences, Shiraz P.O. Box 71468-64685, Iran; 4 Department of Mechanical Engineering, Australian University (AU)-Kuwait, Mishref, Safat 13015, Kuwait; 5 Center for Infrastructure Engineering, Western Sydney University, Penrith, NSW 2751, Australia

**Keywords:** Zinc Oxide nano particles, *Chlorella vulgaris*, microalgae, biosynthesis, lyophilization

## Abstract

The rampant increase in antibiotic resistance has created a global barrier to the treatment of multidrug-resistant infections. Biogenic synthesis of nanomaterials is a novel approach to producing nanostructures with biological resources. Algae are known to be clean, nontoxic, cost-beneficial, and environmentally acceptable. *Chlorella vulgaris* is a popular microalga for its broad applications in food, supplements, pharmaceuticals, and cosmetics. In this study, we used *Chlorella vulgaris* biomass lyophilized powder as our green resource for the biosynthesis ZnONPs. *Chlorella vulgaris* culture was harvested at the end of the logarithmic phase, and the biomass was lyophilized. ZnONPs were synthesized using lyophilized biomass and 20 mM zinc acetate dihydrate at a temperature of 70 °C and continuous stirring in a water bath overnight. At the end of the reaction, UV–Vis absorption of colloidal suspension proved the synthesis of ZnONPs. The physicochemical characteristics of nanoparticles were analyzed using FTIR, DLS, TEM, and XRD. Based on FTIR spectra. The antibacterial activity of green synthesized nanostructures was evaluated against methicillin-resistant staphylococcus aureus (MRSA) and vancomycin-resistant enterococci (VRE). The synthesized ZnONPs have oxygen-containing groups on the surface that show the synthesized nanoparticles’ stabilization. The Zeta potential was −27.4 mV, and the mean particle size was measured as 33.4 nanometers. Biogenic ZnONPs produced in this method have a notable size distribution and excellent surface energy, which can have vast applications like antimicrobial potential in pharmaceuticals as topical forms. Additionally, in order to evaluate the antimicrobial activity of ZnO nanoparticles, we used MRSA and VRE strains and the results showed the anti-MRSA activity at 400 and 625 μg mL^−1^, respectively. Thus, these biogenic ZnO nanoparticles revealed a substantial antibacterial effect against multidrug-resistant pathogens, associated with several serious systemic infections, and have the potential as an antimicrobial agent for further study.

## 1. Introduction

The problem of nosocomial infection is the burden of multidrug-resistant (MDR) microorganisms, which is a major threat to public health. Hospitals can be considered sites of antimicrobial resistance development and factories for the production, circulation, and promotion of antimicrobial resistance [1,2]. The added national cost associated with MDR in hospitalized patients with resistant infections is about US$2.39 to 3.38 billion per year [3]. Methicillin-resistant *Staphylococcus aureus* (MRSA) and Vancomycin-resistant *Enterococcus* (VRE) are the major nosocomial human multidrug-resistant pathogens. About 40–60% of human *S. aureus* samples isolated from hospitals are resistant to methicillin. The situation becomes catastrophic when current reports indicate that a significant number of MRSA strains cannot be treated even with vancomycin, one of the few possible choices to treat these strains, and it even leads to the production of VRE [4]. Morbidity and mortality rates due to MRSA and VRE strains are continuously growing; therefore, many studies have urgently focused on finding a way to control these infections. The zinc oxide nanoparticles (ZnONP) exhibited strong antimicrobial activity against pathogenic microorganisms and their shape are vital parameters related to pharmacological and toxicological responses [5].

ZnONP previously was used for insulin synthesis, storage, and secretion, so being an antidiabetic agent and alleviating diabetic complications is one of the ZnONPs potentials [6]. ZnONPs can trigger the immunotoxicity biomarkers, cytokines, and chemokines, activate the innate immune response, increase the uptake of antigens and regulate the cytokine network [7]. Since ZnONPs cannot pass through the skin and solve in an aqueous solution as ions, they can have roles in hard tissue reconstitution and composite scaffolds for skin tissue engineering as wound dressing materials [8].

The mechanochemical and chemical methods may have several disadvantages for example clear environmental implications and causing toxicity in cells and living tissues [9]. However, by performing appropriate modification process these methods can meet principles of green chemistry. In recent years, algae have been applied more for producing silver and gold nanoparticles than ZnONPs [10]. However, *Chlorella vulgaris* is identified as a desirable choice for producing ZnONPs. Large-scale culturing of *C. vulgaris* causes environmental pollution because of its large quantity of a culture supernatant as waste stream production. Carbohydrates, primary secretory bioactive compounds in supernatant culture, can be used in ZnONPs synthesis and affect the particle size and morphology. It is more beneficial than polyethylene glycol as a shape controller because this method uses the waste material generated from the production of *C. vulgaris* biomass [11]. There are several soluble polysaccharides, and extracellular proteins may be the active compounds in *C. vulgaris* cell extract, benefitting the bioaided fabrication of metal nanoparticles [12,13,14]. Functional groups in protein residues primarily contribute to manufacturing metal nanostructures [12].

In this study, we used lyophilized powder of *C. vulgaris* to produce ZnONPs from the primary substrate via green synthesis. We measured this lyophilized powder’s amount of protein, carbohydrate, and lipid to evaluate the biochemical products and metabolites of microalgae in ZnONPs biosynthesis. The proposed approach may lead to distinctive nonaccumulated nanoparticles. Two nosocomial multidrug-resistant (MDR) bacterial strains are examined in this study under the effect of ZnONPs.

## 2. Material and Methods

### 2.1. C. vulgaris Culturing Conditions

*C. vulgaris* was obtained from the Microalgal Culture Collection of Shiraz University of Medical Sciences (MCCS, Shiraz, Iran). Inoculation of *C. vulgaris* cells (10^7^ cells mL^−1^) was done in a BG-11 broth medium for culturing and maintaining blue cyanobacterial and freshwater algal species, followed by incubation at 28 °C in a 16 h-light/8 h-dark cycle. BG-11 broth medium is made of different materials bought from Merk, Darmstadt, Germany. 

The microalgal growth trend was measured using the direct cell counting method. A Neubauer hemocytometer was used for cell counting within 60 days. The sampling procedure was performed every three days for this 60-day experiment. With the logarithmic growth phase (after incubating for 20 days), the culture was subjected to centrifugation (4000× *g*, 6 min) to harvest the supernatant. It was then washed twice with normal saline solution (Shiraz Serum, Shiraz, Iran) (0.9% *w*/*v*) and centrifuged with the described conditions. The resultant lucid and colorless solution was utilized for the lyophilization process. It was refrigerated at −20 °C overnight and then lyophilized for 48 h. The obtained powder was homogenized using mortar and pestle. The product was stored at −20 °C for synthesizing the ZnO nanoparticles.

### 2.2. Biomass Composition of C. vulgaris

#### 2.2.1. Protein Content 

The total protein content of the studied microalgal strain was determined through the Lowry method [15]. The chlorophyll content was first removed to prevent its interaction with the proteins and for better accuracy of the results. Methanol, (Merk, Darmstadt, Germany) (1 mL) was added to the lyophilized microalgal powder (5 mg) and shaken vigorously, and then was centrifuged at 15,000× *g* for 10 min at 4 °C. The upper green phase was removed, then methanol (1 mL) was added to the obtained pellet and stored at 4 °C overnight. The product was sonicated for 3 min at 20 kHz and centrifuged at 15,000× *g* for 10 min at 4 °C. Then the supernatant was removed. NaOH (Merk, Germany) (0.1 N, 0.1 mL) was added to the pellet and stored at room temperature (25 °C) for 1 h, then centrifuged at 15,000× *g* for 20 min. The obtained supernatant was used for the analysis of protein. Next, trichloroacetic acid (Merk, Darmstadt, Germany) (25%) was added to the remaining pellet and stored on the ice for 30 min, then centrifuged at 15,000× *g* for 20 min at 4 °C. The obtained pellet was washed using trichloroacetic acid (10%) and centrifuged at 15,000× *g* for 20 min at 4 °C. NaOH (1.0 N, 0.2 mL) was added to the precipitated proteins and vortexed. The Lowry indicator (1 mL) was added and mixed gently.

In comparison, the Folin–Ciocalteu phenol reagent (Sigma Aldrich, St. Louis, MO, USA) (0.1 mL) was added to the solution, stored at room temperature for 35 min, and then the absorbance was measured at 750 nm wavelength. The protein content of the lyophilized biomass was measured using the calibration curve. A calibration curve was provided using bovine serum albumin (BSA) (Sigma Aldrich, Darmstadt, Germany) at 0.0 to 1.0 mg mL^−1^ concentration, and the optical density was read using the spectrophotometry method at 750 nm.

#### 2.2.2. Lipid Content 

The lipid content of *C. vulgaris* cells was quantified through the sulpho-phospho-vanillin (SPV) method. A standard curve (0.0–0.3 mg) of olive oil from the Food and Drug Organization of Shiraz University of Medical Sciences was used as the control. Bligh and Dyer method was employed to extract lipids. Briefly, 5 mL of isopropanol (Merk, Darmstadt, Germany) was added to 5 mg of lyophilized microalgal biomass. It was then vortexed and stored in a water bath at 90 °C for 15 min. Chloroform-methanol solution (Merk, Darmstadt, Germany) (2:1, 15 mL) was added to the pellets. Butylated hydroxytoluene (0.01 *w*/*v*) was used as the antioxidant, and the solution was centrifuged at 2000× *g* for 5 min. The supernatant was collected and dissolved in a mixture of chloroform (5 mL)/distilled water (4 mL). KOH (Merk, Darmstadt, Germany) (0.88% *w*/*v*, 5 mL) was added and mixed for 30 min, then centrifuged at 2000× *g* for 5 min. The aqueous phase was collected and stored at 90 °C for 10 min. Sulfuric acid (Panreac, Barcelona, Spain) (98%, 5 mL) was added to the extracted lipids and kept at 90 °C for 20 min. The lipid content was characterized at 540 nm using the spectrophotometry method.

#### 2.2.3. Carbohydrate Content 

The total carbohydrate contents of the studied strain were determined using the 3-methyl-2-benzothiazolinone hydrazone (MBTH) (Merk, Darmstadt, Germany) method [16], where the glucose solution (Merk, Darmstadt, Germany) (0.0–0.5 mg mL^−1^) was used as the standard to draw a calibration curve. Sulfuric acid (72%, 0.1 mL) was added to the lyophilized biomass powder (5 mg). After 30 min storing at 60 °C, 1.4 mL distilled water was added to each microtube. It was boiled at 121 °C for 60 min and then centrifuged at 15,000× *g* for 20 min. For the next step, the supernatant was used. It was diluted with distilled water (250 µL, 1:1) and 0.5 mL NaOH (0.5 M) was added and shaken vigorously. MBTH solution was added to 0.5 mL of the samples and stored at 80 °C for 15 min. HCl (Merk, Darmstadt, Germany) (0.25 mM, 1 mL), containing ferric ammonium sulfate (Sigma Aldrich, Madrid, Spain) (0.5 *w*/*v*) and sulfamic acid (Merk, Darmstadt, Germany) (0.5 *w*/*v*), was added to the samples, along with Distilled water (2.5 mL). The carbohydrate content was determined at 620 nm using the spectrophotometry method.

### 2.3. Synthesis of ZnO Nanorods

The ZnO nanorods were synthesized at ambient temperature through a managed reaction. In the first stage, 1 g of Zn(OAc)_2_·2H_2_O (Sigma Aldrich, St. Louis, MO, USA) was dissolved in 140 mL of *C. vulgaris* culture supernatant. Afterward, 1.5 mL of ammonium hydroxide (Merk, Darmstadt, Germany) (NH_4_OH, 25%) was poured dropwise onto the mixture while the solution was constantly refluxed at 80 °C. After 6 h, the product was rinsed with deionized water, followed by additional reflux for an extra 9 h. Finally, the fabricated particles were rinsed repeatedly with deionized water and desiccated at 60 °C in an oven for a period of 24 h.

### 2.4. Characterization of ZnONP

The functional groups of lyophilized algal biomass and the presence of these biological functional groups on ZnONP were verified using the Fourier transform infrared spectroscopy (FT-IR, Vertex 70, Bruker, Berlin, Germany) ranging from 400 to 4000 cm^−1^. For preparation, the lyophilized algal powder and green synthetized ZnNP were blended with potassium bromide (KBr) powder and pressed into tablets and the IR was measured at room temperature.

X-ray diffraction pattern for the powdered green synthetized ZnONPs was carried out using an X-ray diffractometer (XRD, D800, Siemens, Munich, Germany) that were recorded on normal focus diffractometer with Cu Kα radiation at voltage of 30 kV, current 15 mA and wavelength of *λ* = 1.0154 Å in the scan range of 2*θ* = 20–90°. The morphology, topology, and dimension of the manufactured ZnONP were examined through scanning electron microscopy (SEM, 10 kV, Tescan Vega 3, Czech Republic). Lens-mounted DBS and LVD offer the best selection of information and image optimization. The beam landing energy goes down from 30 KeV to 50 ev and has a resolution of 1.4 nm at 1 kV and 1 nm at 15 kV.

Transmission electron microscopy (TEM, EM900, Zeiss, Aalen, Germany) images with the magnification of 10,000 kx were used to evaluate the morphology of algal synthesized nanostructures. For measuring the size of particles, we used TEM image and image size software. In order to examine the particle’s size, we calculated the size of 100 distinct nanoparticles and measure the average size using the obtained size.

The net surface charge of the nanostructure was measured using a laser particle size analyzer (Zetasizer Nano ZS90, Malvern, England). UV–Vis absorption spectra were obtained by a T80+ UV–Vis spectrometer (PG Instruments, Sydney, Australia) within 200 to 800 nm.

### 2.5. Antimicrobial Activity

For the antimicrobial property, the produced nanorods were assessed through the microdilution method, which was designed based on the protocols of the Clinical and Laboratory Standards Institute (CLSI, Wayne, PA, United States) [17]. The methicillin-resistant *Staphylococcus aureus* (MRSA) and vancomycin-resistant *Enterococcus* (VRE) isolates were procured from Namazi Hospital in Shiraz (Shiraz, Iran). The resistance potential of the bacterial samples was tested before antimicrobial testing. Twofold serial dilutions of ZnONP were made using Mueller–Hinton Broth (MHB, sigma Aldrich, Madrid, Spain) medium from 800 to 25 µg/mL. Each dilution was poured into a 96-well microplate containing bacterial suspension, followed by incubation at 37 °C for 24 h. An ELISA reader (Biotek, Winooski, VT, USA) was used to measure the optical density of the microplates at a wavelength of 600 nm. By definition, the MIC90 was considered the value that suppressed 90% of the bacterial growth against the control group. The culture media and those inoculated with bacterial strains were assumed as negative and positive control groups, respectively. The microorganism viability was calculated using the below equation.

% microorganism viability = (OD_sample-blank_)/(OD_control-blank_) × 100

### 2.6. Statistical Analysis

The significant differences in the results (*n* = 3) were determined using a standard *t*-test. The statistical difference level of 5% was regarded as significant. The IBM SPSS software version 23.0 (IBM Corp., Armonk, NY, USA) was used for the statistical analysis.

## 3. Results and Discussion

### 3.1. Growth Measure

**Figure 1:**
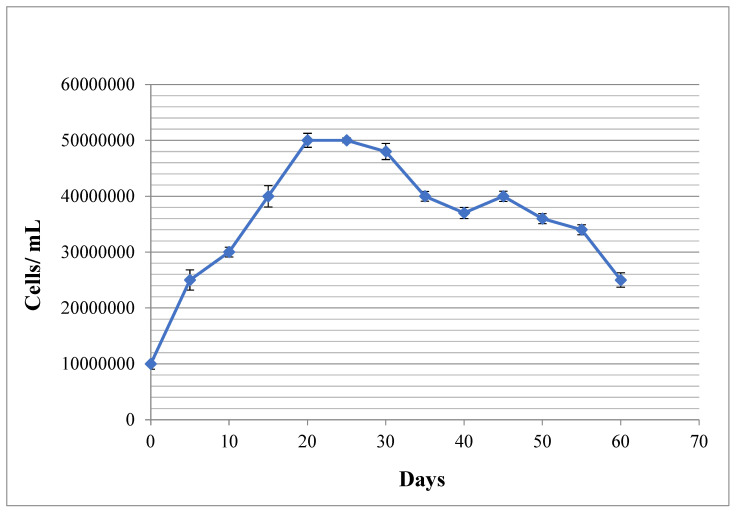
Microalgal growth trend during 60 days of experiment using direct cell counting method. The bars on each dot represent standard deviation of three experiment.

This 60-day experiment was performed three times, and the average values were measured and used for data analysis. Figure 1 shows the growth profile of *C. vulgaris* strain with a typical sigmoidal pattern. The logarithmic phase lasts for 20 days, then the stationary phase is initiated. The death phase was started on the 28th day. A secondary growth phase was seen on the 48th day due to the cannibalistic events. The experiment was done in triplicates and the standard deviations have been shown as bars in the Figure 1.

### 3.2. Composition Analysis of Biomass

**Figure 2:**
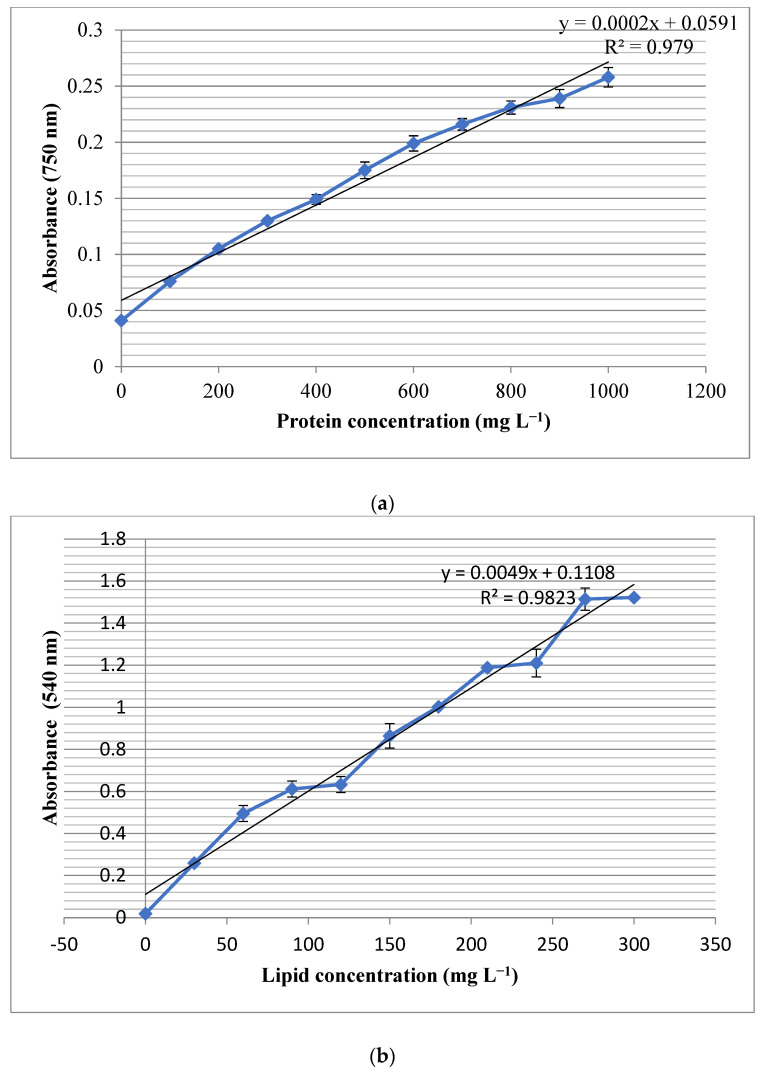

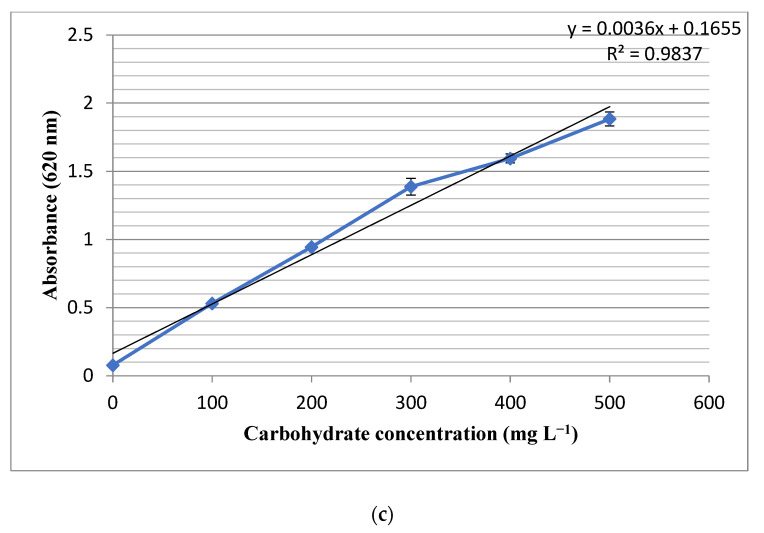
Standard curves and R^2^ values for (**a**) protein, (**b**) lipid and (**c**) carbohydrate determination. The bars on each dot represent standard deviation of three experiment.

The results showed that the total protein content of the studied strain was 0.61 mg in 1 mg of the lyophilized powder. The measured R^2^ for the growth pattern was found to be 0.979, which indicated the accuracy of the observed results (Figure 2a). Moreover, the studied strain contained 0.13 mg lipids in one mg of biomass and the experimental R^2^ value of 0.9823 (Figure 2b). Moreover, the total carbohydrate contents of the studied cell line were measured as 0.14 mg in 1 mg of the lyophilized biomass powder, and the R^2^ value of 0.9837 (Figure 2c). The experiment was done in triplicates and the standard deviations have been shown as bars in the Figure 2a–c.

**Figure 3:**
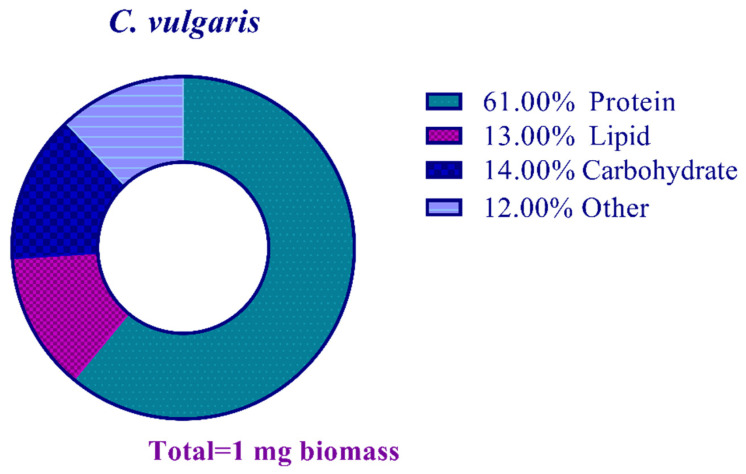
Biomass composition analysis of the studied *C. vulgaris* strain.

There was a total of 0.12 mg mg^−1^ of nucleic acid and other undetermined components in the lyophilized biomass (Figure 3).

**Figure 4:**
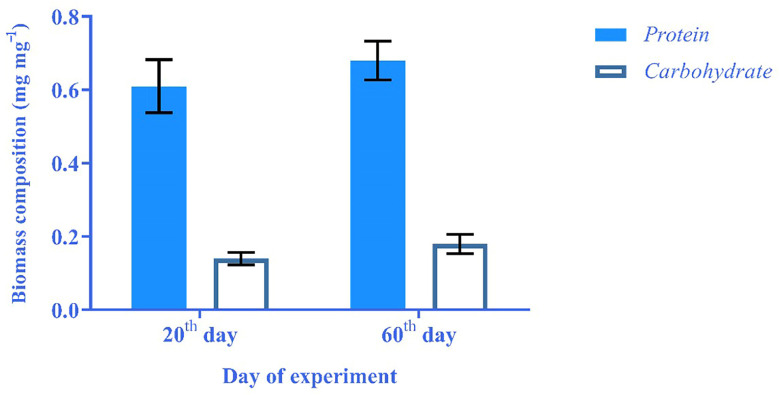
Comparison of protein and carbohydrate contents in 20th and 60th day of experiment.

The obtained results were compared to each other to assess the effects of the growth phase on biomass composition. It was revealed that the protein and carbohydrate content of biomass on the 60th day was 0.07 mg, 0.04 mg higher than the same results on the 20th day of the experiment (Figure 4). The observed phenomenon might be attributed to the cell lysis at the death phase, which led to the release of biochemical components.

### 3.3. Characterization of ZnONP

**Figure 5:**
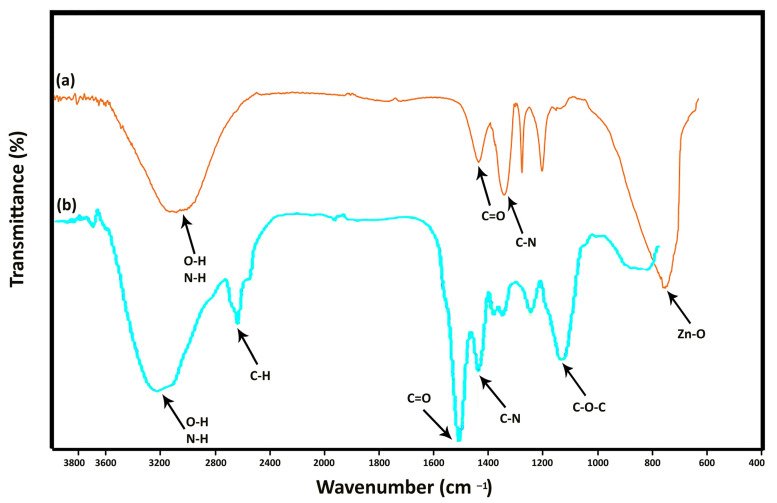
FT-IR spectra of the fabricated ZnO nanorods. (**a**) is representing the FT-IR spectra of the algal synthesized ZnONP, (**b**) is representing the FT-IR spectra of *C. vulgaris*.

Figure 5 illustrates the FT-IR spectra of *C. vulgaris* and the algal synthesized ZnONP. In the Figure 5b, the peak at 3330 cm^−1^ was attributed to O-H stretching N-H stretching bands which are due to hydroxyl and amide A groups found in protein contents of microalgae. The absorption peak at around 2800 cm^−1^ was attributed to H-C-H stretching bands founds in lipid/carbohydrate contents. The characteristic band at around 1630 cm^−1^ was assigned to C=O stretching founds in proteins of *C. vulgaris*. The band at around 1510 cm^−1^ was assigned to C-N stretching bands of amide II found in proteins. The peaks at 1380 cm^−1^ to 1250 cm^−1^ are assigned to C-H bending modes of methyl groups of carboxylates lipids. Finally, the peak at 1050 cm^−1^ was assigned to C-O-C bands of polysaccharides.

In the Figure 5a, the absorption peak at around 3330 cm^−1^ is assigned to the stretching vibrations of O-H groups. The band at 1630 cm^−1^ is attributed to the stretching vibration of carbonyl groups, while the peak at 1282 cm^−1^ belongs to the C-C bond. The two strong peaks at 1494 cm^−1^ and 1396 cm^−1^ correspond to the nitro groups and C-H bending modes of methyl groups of carboxylates lipids. The peak situated at 580 cm^−1^ is the representative peak of the stretching mode of metal-oxygen bond in spinel structures in ZnO.

These peaks clearly show that ZnO nanostructures have been synthesized and there are molecules related to *C. vulgaris* microalgae on its surface. This characterization shows the success of the green synthesis process of ZnONP. The results of this characterization are consistent with the results of Kahzad et al. who synthesized CuFe_2_O_4_@Ag nanocomposite using *C. vulgaris* microalgae [18].

**Figure 6:**
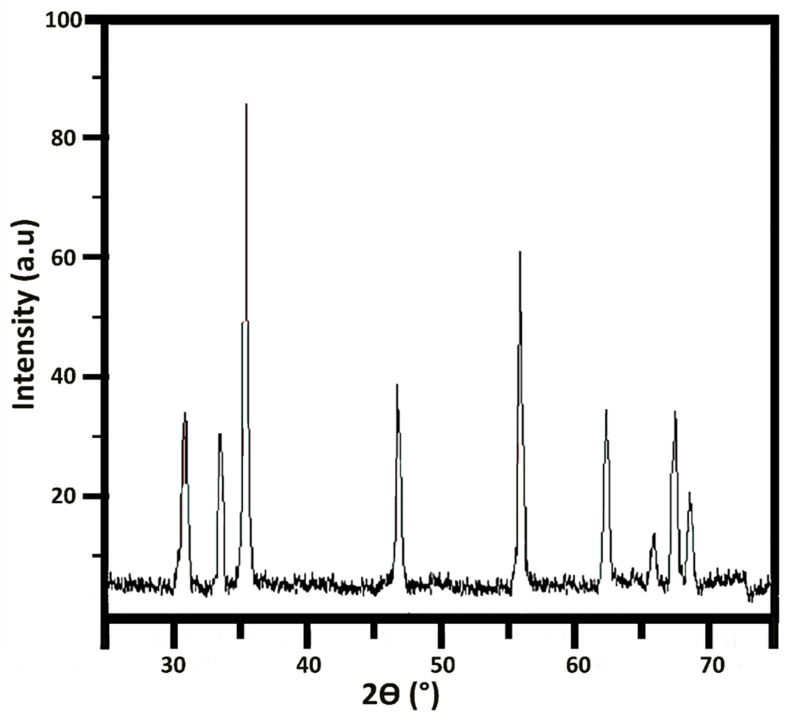
X-ray diffraction of the ZnO nanorods.

Figure 6 depicts the X-ray diffraction pattern of the formulated nanorods that shows an excellent crystalline structure. Despite the crystalline structures, some spherical structures in synthesized nanoparticles show the existence of an amorphous phase in nanorods. The crystallite size was calculated as 3.4 nm through the Scherrer calculator using X’Pert HighScore version 3.0.5 (Philips, Eindhoven, The Netherlands).

**Figure 7:**
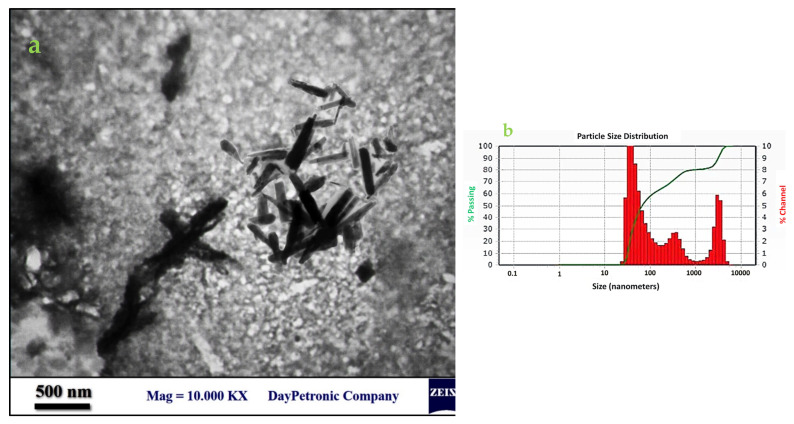
TEM image of the ZnO nanorods (**a**) and the size distribution chart using particle size analyzer (**b**). Average particle size distribution plot (red) size vs. % channel (green) size vs. % passing. % Passing-Cumulative values of each size of particles from 0 to 100 %; % Channel-Size distribution values for each size of particles.

The size distribution and morphology of the ZnO nanorods were analyzed via TEM (Figure 7). The manufactured nanostructures were rod-shaped with an average length and width of 633.77 nm and 75.61 nm, respectively, and the aspect ratio (described as the length to width ratio) of 8.38. Despite a relatively normal distribution in the length of nanorods, the algal synthesized nanoparticles had no uniform form and size. The zeta potential of algal synthesized ZnO nanorods was −27.4 ± 0.72. This negative surface charge was due to the net charges of surface biological compounds, especially protein residues and their corresponding hydroxyl and carboxyl groups in biological solutions.

**Figure 8:**
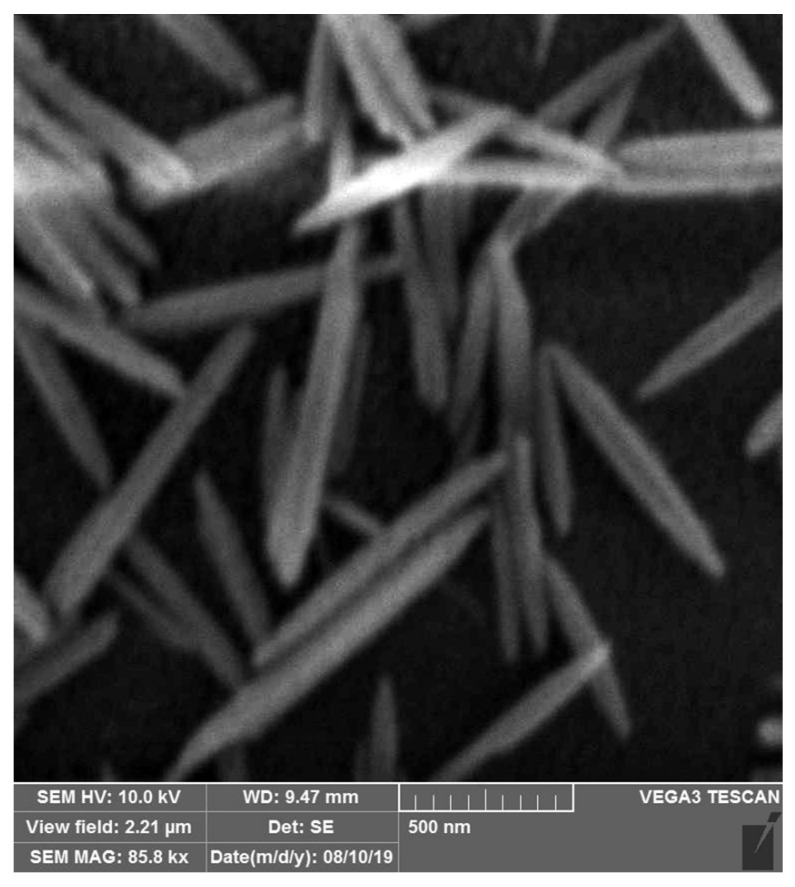
SEM image of ZnO nanorods.

Here, the extracellular substances from *C. vulgaris* were utilized for the bioaided fabrication of ZnO nanorods. As shown in the SEM image (Figure 8), the resultant ZnO nanorods were particles with distinct separation due to the application of extracellular substances from *C. vulgaris*, which represents that most of the ZnO nanorods have a crystalline structure. Thus, the quality of the manufactured ZnO nanorods represents the successful implementation of this method. Our findings were supported by those reported recently, indicating that the biological compounds from *C. vulgaris* had a shape-determining role [14]. Two distinct classes, cell extract compounds, and extracellular substances, can comprise two groups of biological macromolecules from *C. vulgaris* with different structures and specifications [14,19]. Previously, we demonstrated that natural biological macromolecules such as intermediate filament protein skeins and mucin vesicles played an essential role in designing three-dimensional (3D) nanoscaffolds [20]. A similar observation in the bioaided fabrication of ZnO nanoparticles with *C. vulgaris* cell extract has been reported by Zhang et al. [21]. Their finding showed that *C. vulgaris* extract could be used as an additive to the synthesizing reaction, resulting in plate-shaped nanostructures.

According to a previous study on the secretive compounds of *C. vulgaris*, the production of silver nanoparticles was effectively mediated by carbohydrates. The spherical nanostructures were formed uniformly with an isotropous growth model. Likewise, secretive compounds from *C. vulgaris* were used to synthesize FeOOH nanospheres in a controlled manner. This experimental finding demonstrated that the isotropous growth of metal nanocrystals was not always driven by secretive compounds from *C. vulgaris*. Other reports noted a form-determining role of carbohydrate polymers in the growth of ZnO nanocrystals. Mainly, xanthan gum and PEG were found to be efficacious form-determining factors. Based on these investigations, ZnO nanoparticles were transformed into rod-shaped structures upon increasing PEG concentration, suggesting the formation of ZnO nanoparticles with no use of regulatory agents.

**Figure 9:**
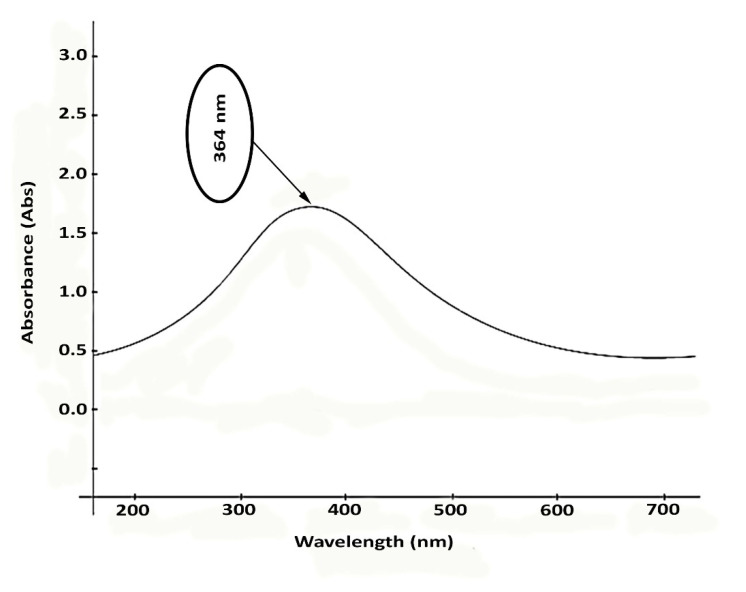
UV absorbance of the microalgal synthetized ZnO nanorods.

As seen in the spectra of Figure 9, the ZnO nanorods showed their potentiality in absorbing UV–Vis irradiation. An absorption peak at 364 nm for the fabricated nanorods represented the absorbing action of ZnO nanostructures. This essential feature uniquely characterizes ZnO particles, making these materials appropriate for producing pharmaceutically valued compounds (e.g., sun blockers). As an effective sunscreen, ZnO particles can protect against the negative impacts of UV radiation. In contrast, the protection against wideband UV can generally be reached by combining ZnO with titanium dioxide particles (TiO_2_).

The UV–Vis spectrum shoulder in Figure 9 confirms that the manufactured nanorods can effectively act toward UVB (290–320 nm) and UVA (320–400 nm) lights. The UV irradiation impact influences the biological function of ZnO nanostructures. ZnO is highly efficient photo catalytically, and its interplay with bacterial cells is improved dramatically by its strong capability of absorbing UV radiation, which facilitates the suppression of cellular growth or death through the formation of reactive oxygen species (ROS).

**Figure 10:**
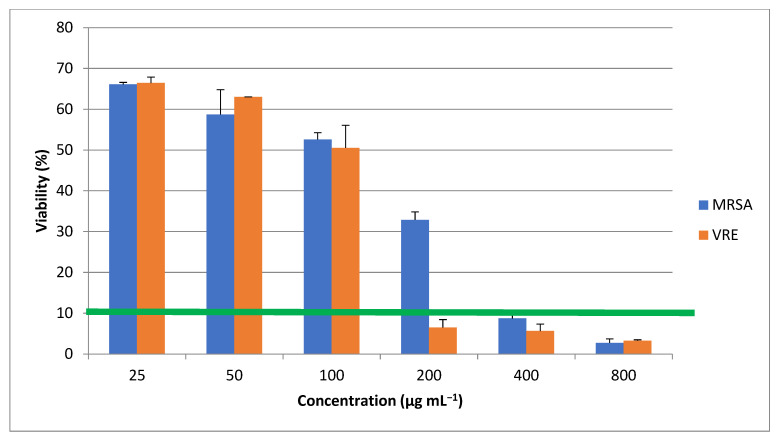
Antimicrobial effects of microalgal synthetized ZnO nanorods. * shows the *p* value ≥ 0.05 and MIC threshold (green line) at 10% show the lowest concentration in which nanoparticles inhibit the microorganisms’ growth more than 90%.

The ZnO nanorods were assessed in terms of their antimicrobial property toward MRSA and VRE bacterial strains, as illustrated in Figure 10, that depended on their concentrations, 200 µg mL^−1^ against VRE and 400 µg mL^−1^ against MRSA (Table 1). The antimicrobial property declined gradually upon increasing the concentration, possibly because the nanorods aggregated interestingly. These findings agreed with other studies that reported an elevated antimicrobial property associated with increasing nanoparticle concentrations [22,23,24,25,26]. Compared with single particles, the aggregations may differ in form and size. The antibacterial activity of the nanorods might decline when they are agglomerated because there are reports of nanostructure biological activity dependent upon both the shape and size of the particles [27]. A significantly higher antimicrobial property is sustained by certain scales, with less potentiality for greater particles, e.g., micron sizes, than their nanoscaled counterparts. As it can be seen in Figure 10, the produced ZnO nanoparticles have significant difference (*p* value ≥ 0.05) at 200 µg mL^−1^ which means they have more considerable antimicrobial effect on MRSA rather than VRE. The MIC threshold at 10% defines the significant limit of nanoparticles effectiveness between MRSA and VRE. The 0.05 ≥ *p* value and the MIC threshold demonstrate the concentration in which nanoparticles’ concentration can inhibit the microorganism growth more than 90%. 

The ZnO nanorods influenced the cell survivability of both MRSA and VRE bacterial strains. Gram-positive bacteria are more susceptible to the antibacterial function of ZnONPs as their cell wall structure, cell physiology, metabolism, and exposure level are different from Gram-negative [28]. Even so, the zone of inhibition was determined in the above investigations for quantifying the antimicrobial property, while absorbance levels were measured here to evaluate the antibacterial function. In other reports, comparisons were made for the antimicrobial impacts of ZnO nanostructures on *S. aureus* and *E. coli*. At the same time, we examined two nosocomial multidrug-resistant (MDR) bacterial strains in this study. 

Previous studies showed MIC and MBC values of ZnO nanoparticles against isolated MRSA strains were 312.5, 625, and 1250 μg mL^−1^, indicating good bactericidal activity [29]. These results agree with the present outcomes, except we showed the anti-MRSA activity at 400 and 625 μg mL^−1^. Some other studies also revealed the inhibitory potential of ZnO nanomaterials against MDR pathogens in a concentration-dependent manner [30,31].

There is a considerable variation in the antibacterial function of ZnO nanostructures [4,32,33]. Although many research outputs presented evidence of downgrading the microbial growth and metabolism by ZnO nanostructures, Ebrahiminezhad et al. synthesized xanthan gum-coated microstars with biocompatibility, exhibiting no antimicrobial properties [34]. The particle size was primarily responsible for differences in the antimicrobial function of ZnO particles [33]. Biocompatible coatings and other types may sometimes influence the antimicrobial features of resultant particles [2,25].

ZnO nanostructures can proposedly exert their antimicrobial function in multiple ways, as they can interact with either the surface or the core of bacterial cells to induce a variety of antibacterial modes of action [35]. There are probably three modes of action for the antibacterial property, including generating ROS, releasing zinc ions (Zn^2+^), and changing the penetrability of bacterial cellular membranes. ROS formation presumably is the mode of action primarily involved in the antibacterial function of ZnO nanostructures [36,37]. Vital cellular constituents (e.g., DNA, phospholipids, and proteins) are targeted and destroyed by ROS, leading to either suppression of cellular growth or death. The antibacterial action of ZnO nanostructures is also exerted by releasing Zn^2+^ into adjacent environments as a further important reservoir. This is because the discharged Zn^2+^ can bind to biomolecules (e.g., proteins and carbohydrates), disrupt enzyme systems, and inhibit active transportation and amino acid metabolism in bacterial cells, damaging cell survivability [35]. A further possible toxicity induction is penetrating and accumulating nanostructures in the cellular membrane, thereby dissipating the proton motive force and leading to the altered penetrability of the plasma membrane. This gradually releases lipopolysaccharides, membrane proteins, and intracellular agents from the bacterial cell and thus reduces the cell’s survivability [35].

This promising antimicrobial effect of biologically synthesized ZnO nanorods against nosocomial pathogens may prompt the implementation of this biological nanostructure in various pharmacological and other health-related industrial applications such as cosmetics, sunblockers, and food packaging.

## 4. Conclusions

Extracellular substances from *C. vulgaris* synthesized ZnO nanorods by developing a productive approach. In this study we cultured microalgae and evaluated their features in order to produce nanoparticles. Regarding to their characteristics we found that they have features that can’t be obtained in chemical nanoparticles production such as their protein and carbohydrate contents. The average length and width of the produced particles were 150 nm and 21 nm, respectively. The fabricated nanorods could block radiations at UVB and UVA levels which is helpful for sun protectors. Assessment of the antimicrobial property of the ZnO nanorods was also done toward both Gram-positive and Gram-negative bacteria through the microdilution procedure. The ZnO nanorods exhibited prominent antibacterial function, with the best effectiveness established at a concentration of 250 µg mL^−1^. With antibacterial features, the manufactured ZnO nanorods can be applied in food packing, the therapeutic area, and various other antimicrobial utilizations. It should be mentioned that the extracellular substances from *C. vulgaris* can be obtained as culture supernatant as a microalgal spinoff of plants that produce biomass. Such a low-cost substance can lower the expenses of this procedure, thereby providing an alternate method with better profitability than chemicals generally utilized in synthesizing nanostructures. Some studies have indicated the possibility of using extracellular vehicles (EVs) for drug delivery. Recently a new category of EVs has been exploited as nanoalgosomes which exhibit various pharmaceutical and biotechnological potentials [38,39]. Such studies may help develop recent technologies such as virosome-based nanovaccines and other newly developed biomacromolecules [40].

Further studies could be performed on our studied microalgal strains for possible nanoliposome production. Furthermore, the fabricated nanostructures are significantly affected by concentrations of controllers. Hence, applying diluted culture supernatant results in obtaining nanostructures with different specifications, which represents an exciting topic for future investigations.

## Figures and Tables

**Table 1:** MIC values of ZnONP against MRSA and VRE.

	MRSA	VRE
ZnONP (µg mL^−1^)	400	200

## Data Availability

All the Data obtained in this study are available in the context.

## Abbreviations

BSA: bovine serum albumin; CLSI: clinical and laboratory standards institute; DLS: dynamic light scattering; EVs: extracellular vesicles; MCCS: Microalgal Culture Collection of Shiraz University of Medical Sciences; MBC: minimum bactericidal concentration; MBTH: 3-methyl-2-benzothiazolinone hydrazone; MHA: Muller–Hinton agar; MHB: Muller-Hinton broth; MRSA: methicillin-resistant *Staphylococcus aureus*; PEG: polyethylene glycol; SEM: scanning electron microscopy; SPV: sulfo-phospho-vanillin; TEM: transmission electron microscopy; VRE: vancomycin-resistant enterococci; XRD: X-ray powder diffraction; ZnO: zinc oxide.
